# Validation of Italian version of Brace Questionnaire (BrQ)

**DOI:** 10.1186/1748-7161-8-13

**Published:** 2013-08-20

**Authors:** Angelo G Aulisa, Vincenzo Guzzanti, Marco Galli, Carmen Erra, Giorgio Scudieri, Luca Padua

**Affiliations:** 1 Orthopaedics and Traumatology Division, Bambino Gesù Children’s Hospital, Institute of Scientific Research, Piazza San Onofrio 4, 00165 Rome, Italy; 2 Department of Orthopaedics, University of Cassino, Strada Folcare, 4, 03043 Cassino, FR, Italy; 3 Don Carlo Gnocchi Foundation, Piazzale Morandi 6, 20121 Milan, Italy; 4 Department of Orthopedics and Traumatology, Catholic University of the Sacred Heart, University Hospital “Agostino Gemelli”, L.go A. Gemelli 1, 00168 Rome, Italy; 5 Department of Neurosciences, Institute of Neurology, Chatolic University of Sacred Heart, University Hospital “Agostino Gemelli”, L.go A. Gemelli 1, 00168 Rome, Italy

**Keywords:** BrQ, Quality of life, Brace, Adolescent idiopathic scoliosis, Brace questionnaire

## Abstract

**Background:**

Brace questionnaire (BrQ) is a tool used to evaluate Health Quality of Life (HRQoL) in patients with Adolescent Idiopathic Scoliosis (AIS) that undergo bracing treatment. The BrQ has not been translated and validated for Italian-speaking patients with AIS. The aim of the study was to perform a trans-cultural validation of BrQ to be used in an Italian speaking population.

**Methods:**

Translation into Italian (I-BrQ) and back translation to the original Greek (G-BrQ) was performed. The final I-BrQ was then analyzed for Italian cultural characteristics and no inconsistencies were found. After that, construct validity was measured analyzing the I-BrQ relationship with 1) Scoliosis Research Society-22 patient Questionnaire (SRS-22), in order to evaluate the relationship with another patient-oriented questionnaire not focused on brace therapy; 2) Cobb degree scale, in order to evaluate the relationship with the magnitude of the curve. Reproducibility was also tested.

**Results:**

Translation of the G-BrQ into Italian was successful and back-translation to Greek corresponded well with the original Greek version.

Global I-BrQ correlated strongly with SRS-22 (r = 0.826; p < 0.001). Almost all sub scores from each I-BrQ domain strongly correlated with the single domain scores of SRS-22. Only two I-BrQ sub scores weakly inversely correlated with Cobb degree value.

Reproducibility was good (Spearman-Brown coefficient value was 0.943; p < 0.05).

**Conclusions:**

Trans-cultural validation in Italian language showed the validity and reliability of the I-BrQ.

## Background

World Health Organization defined (1948) that “health is a state of complete physical, mental and social well-being and not just the absence of disease” [1]. This concept produced a great change in the health conception and the introduction of a new paradigm, called “bio-psycho-social model” [2]. It included the patients’ perception and promoted the concept of Quality of Life (QoL).

QoL is defined by the World Health Organization as “individuals’ perception of their position in life in the context of the culture and value systems in which they live and in relation to their goals, expectations, standards and concerns” [3]. HRQoL is an aspect of QoL related to personal health. As a way of measuring QoL, self-administered questionnaires have become the most commonly used means to assess the patients’ perspective of health. Patient-oriented measures using such questionnaires have added a new dimension to clinical outcome evaluation.

They are important both for assessing individual patients in the daily clinical activity and for international comparisons, multi-center trials and other types of collaborations. There are two main types of instruments available for this purpose: 1) generic questionnaires, such as the most commonly used health survey Short Form-36 item questionnaire (SF-36) [4] and 2) disease specific questionnaires, which focus on specific aspects of impact in daily life due to a particular disease. Disease specific questionnaires have proven to be more sensitive than generic patient-oriented counterparts [5].

In order to be suitable for research and clinical practice, self-administered questionnaires must undergo though a validation process to evaluate reliability and validity [4]. Moreover a validated questionnaire, in order to be used in a different language speaking population, has to be subject to a process called trans-cultural validation [6].

Adolescent Idiopathic Scoliosis (AIS) is a frequent pathology (prevalence of 1% to 2%) that consists in a three-dimensional deviation of the spinal axis and affects young adults between 10 and 18 years old [7].

Although scoliosis is usually a mild condition and is rarely associated with severe complications, scoliosis itself and its treatment can largely affect patients’ quality of life, especially adolescents [8].

In a study on women with scoliosis in which HRQoL was analyzed with SF-36 and Berner Questionnaire for Well-Being (BFW), it was showed that juvenile patients were unhappier with their lives, had more physical discomfort, lower self esteem and greater depression scores compared with the healthy population sample [9].

Freidel et al., using generic questionnaires such as SF-36, BFW, and the Trait-form of STAIK (a translation of the State Trait Anxiety Inventory: STAIC), showed how idiopathic scoliosis in children, adolescents and adults can be regarded as a risk factor for worsening of HRQoL. Their results were independent from scoliosis magnitude measured with Cobb degree scale [10].

Some papers assessed HRQoL in patient with scoliosis but as they utilized generic questionnaire they perceived the need to use disease specific questionnaires [11-13].

As QoL and HRQoL became one of the major concerns in patients with idiopathic scoliosis, disease specific questionnaires such as Scoliosis Research Society-22 patient questionnaire (SRS-22) were developed in order to assess outcome in adolescents with AIS [14]. However, SRS-22 does not include any specific question on the influence of conservative bracing therapy on HRQoL of adolescents with AIS.

Conservative treatment with bracing was reported to produce stress [15-18], although some authors found no difference in QoL of patients with scoliosis treated with brace [19,20].

In order to determine HRQoL of adolescents with idiopathic scoliosis that undergo bracing treatment, new questionnaires were developed, such as Brace Questionnaire (BrQ) and Bad Sobernheim Stress Questionnaire (BSSQ) with specific focus on HRQoL aspects related with bracing therapy in patients with AIS [21,22].

BrQ was designed to investigate eight specific domains (general health perception, physical functioning, emotional functioning, self-esteem and aesthetics, vitality, school activity, bodily pain and social functioning) [21].

In order to be used as outcome measure in Italian speaking population this questionnaire must be translated and culturally adapted. It must then be validated against the original version. This paper concerns a previously studied sample of patients in which quality of life and therapeutic effects of bracing were evaluated [23]. For that study a preliminary validation of BrQ was performed in 34 patients.

## Methods

The aim of this study was to perform a trans-cultural adaptation of BrQ for use in an Italian speaking population. For translation and cross-cultural adaptation we followed widely accepted guidelines described by Guillemin [6,24]. The validation steps are described as follows.

### Translation

The original BrQ questionnaire was developed in Greek (G-BrQ). We assumed the published G-BrQ and we used it for translation. A physician and a professional Italian-Greek translator, whose first language is Italian, initially translated the G-BrQ into Italian. Another physicians, whose first language is Italian, complete and correct translation from the G-BrQ to Italian. As recommended by Guillemin, a back-translation to Greek was then performed from a Greek physician, working in Italy, in order to check for inconsistencies with the original text.

### Validation methodology

Validity is an index of how well a test measures what it is supposed to measure. Criterion validity is the correlation of a scale with a valid, accepted universally acknowledged measure of the trait or disorder under study. When there is no universally acknowledged measure, construct validity is generally sought to demonstrate the validity of a scale. To evaluate construct validity, a theoretical construct is invoked between the attribute under study and the other attributes that are expected to be related. Therefore, the relationship between these attributes is measured [6,24]. Because there is no universally acknowledged measure of perspective of patients with scoliosis, construct validity was sought to support the over-all validity of the questionnaire.

The following outcome measures for construct validity were adopted:

•SRS-22, in order to evaluate the relationship with another patient-oriented questionnaire not focused on brace therapy;

•Cobb degree scale value, in order to evaluate the relationship with the magnitude of the curve.

We then tested reproducibility, or test-retest reliability, which is a measure of stability (the ability of a scale to give the same results when administered on separate occasions) [23]. Reproducibility was evaluated in a group of 34 clinically stable patients by administering the same protocol (I-BrQ, SRS-22), by the same examiner, who repeated I-BrQ after a mean of 5 days with a range of 3–7 days (to reduce the memory effect, the retest version of the questionnaire differed from the first for the order of the questions).

### Sample

The authors approved the final I-BrQ, after consent was obtained. We administered the questionnaire to 108 consecutive Italian patients with AIS (male/female; 16/92; mean age 15.4 years range 9–18) who wore day long the following braces (see below) for at least 4 months.” Diagnosis of AIS was based on clinical features and X-Rays.

Participants presented with single thoracic curves (n = 31), single lumbar curves (n = 12), single thoracolumbar curves (n = 48), or thoracic and lumbar curves (n = 17). The mean age at the time of questionnaire administration was 15.4 ± 0.2 years (range: 9–18 years). The Risser score at the beginning of treatment was 0–2. The mean curve amplitude at baseline was 32.1 ± 1.0° Cobb (range: 18-70°; median: 30.0°), including 11 patients with surgical curves (46-70° Cobb) who had previously refused surgical treatment, and 18.2 ± 1.1° Cobb (range: 0-55°; median: 15.0°) at the time of interview. The difference in Cobb degrees between baseline and the time of questionnaire administration was −13.9 ± 0.7° (range: -30/-15°; median: 14.0°). Concerning the type of used brace, PASB (progressive action short brace) (39 patients) and Lyon brace (58 patients) were adopted; 11 patients wore both type of brace during their treatment.

The combined approach was prescribed in cases of stiff thoraco-lumbar curves to optimize the hump remodeling. These patients were required to wear the Lyon brace when at home and the PASB while outdoors. Full-time (i.e., 22 hrs per day) bracing was prescribed in all cases. Patients with severe systemic disease were excluded.

### Outcome tools

BrQ includes 34 items organized into 8 domains: general health perception (items 1 and 2), physical functioning (items 3–9), emotional functioning (items 10–14), self-esteem and aesthetics (items 15 and 16), vitality (items 17 and 18), school activity (items 19–21), bodily pain (items 22–27) and social functioning (items 28–34). Every item has five possible answers with a score ranging from 1 to 5 depending on the question: “always” (sempre) received a score of 5 in item number 5, 6, 12, 14, 15, 16, 17 while in the remaining items “never” (mai) received a score of 5. The final score is calculated by multiplying each item score by 20 and by dividing the final score by 34. The BrQ summary score may range from 20 to 100, with higher scores meaning better QoL. Sub scores for each domain (see above) can be calculated by dividing the total score of each domain by the number of items that include it [21] (Additional file 1).

SRS-22 comprises 5 domains (Function, Pain, Mental Health, Self-Image, Management Satisfaction/Dissatisfaction) up to a total of 22 items. The score ranges from 1 to 5 points for each item, with a summary score between 22 and 110 [14]. SRS-22 is a more recent version of the Scoliosis Research Society patient questionnaire (SRS-24): this was originally developed to assess outcome in adolescents with AIS before and after surgical treatment [25]. In our study, we used the Italian validated version of SRS-22, from now on called I-SRS-22 [26].

I-BrQ and I-SRS-22, being self administered questionnaire, were filled by the patients. Aid in filling the questionnaires was provided when requested by the patients. The questionnaires were filled in by the patient at the end of the clinical visit. However, in order not to influence the results of the questionnaire, result of clinical examination was not reported to the patient until the questionnaire was completed.

Cobb degree scale was assessed to measure the lateral curvature of scoliosis on a frontal plane by analysing antero-posterior radiographs of the full spine: the curvature is measured by an angle formed by the intersection of two lines drawn perpendicular to the top endplate of the upper most tilted vertebra and the bottom endplate of the lower most tilted vertebra. Cobb degree was calculated at the first clinical examination and at the time of questionnaire filling. Only this last measure was used to assess construct validity.

### Statistical analysis

Statistical analysis was performed to assess reliability and validity. SPSS 18.0 software was used for the statistical analysis.

The first statistical property analysed was “construct validity”, assessed by comparing I-BrQ scores with SRS-22 through Spearman rank correlation coefficient (r). We also evaluated the correlation between I-BrQ scores and Cobb degree value at time of questionnaire filling.

Test-retest reliability was calculated through Spearman-Brown reliability coefficient.

For all tests statistical significance was set at p < 0.05.

## Results

Translation of the G-BrQ to Italian was successful and back-translation to Greek corresponded very well with the original Greek version. The translation followed the original version except for the example included in the original version that we removed, being the questionnaire very clear. The final I-BrQ was then analyzed for Italian cultural characteristics and no inconsistencies were found. The I-BrQ questionnaire is presented in Additional file 1.

Most patients had no problem completing the questionnaire and few patients had slight problems (16). The duration for each patient to complete it was approximately 10/13 minutes. After filing the I-BrQ, the patients were asked, through a non-structured interview, their opinion on the questionnaire and they all considered the questions relevant to adequately describe their daily situation and patients. All patients answered all questions. Mean values, range and standard deviations of total score and sub-scores of I-BrQ and SRS-22 are reported in Table 1.

**Table 1:** Mean values, range and standard deviations of total score and sub-scores of I-BrQ, SRS-22 and Cobb degree

	**Mean**	**Min value**	**Max value**	**Standard deviation**	**25 percentile**	**75 percentile**
**Total score BrQ**	78.7	55.3	96.5	10.4	71.2	87.3
**BrQ General Health perception**	3.5	1.5	5.0	0.9	2.7	4.5
**BrQ Physical Functioning**	4.0	2.0	5.0	0.63	3.7	4.6
**BrQ Emotional Functioning**	3.5	1.6	5.0	0.7	3.0	4.2
**BrQ Self-esteem and aestetics**	3.6	1.0	5.0	1.0	3.0	4.7
**BrQ Vitality**	3.5	1.5	5.0	0.9	3.0	4.5
**BrQ School Activity**	4.4	2.3	7.0	0.7	4.1	5.0
**BrQ Bodily Pain**	4.0	1.7	5.0	0.6	3.5	4.6
**BrQ Social Functioning**	4.0	2.0	5.0	0.7	3.6	4.7
**Total Score SRS-22**	85.8	59.0	107.0	10.7	79.5	94.0
**SRS-22 Function**	19.6	12.0	25.0	2.7	17.5	22.0
**SRS-22 Pain**	21.5	15.0	25.0	2.6	19.5	24.0
**SRS-22 Mental Health**	19.1	8.0	26.0	3.8	16.0	22.0
**SRS-22 Self image**	16.9	8.0	25.0	3.4	15.0	20.0
**SRS-22 Management Satisfaction/dissatisfaction**	8.6	5.0	10.0	1.2	8.0	10.0
**Cobb (at quest time)**	18.6	0.0 ^¤^	55.0	11.0	10.0	25.5

^¤^3 patients had 0 degree at the time of questionnaire fill-in (they had abnormal Cobb degree at first visit but improved up to complete resolution after wearing brace).

No patients made the maximum or minimum score. The points of I-BrQ ranged between a minimum of 55.3 to a maximum of 96.5 (Table 1) with a 25 percentile of 71.2 and a 75 percentile of 87.3. The majority of the population was therefore moved towards high scores. This, similar to the G-BrQ results, could be due to the fact that either the selected patients had a mild form of scoliosis or the questions designed in the original versions were too harsh on how the brace could affect the QoL of these patients.

Table 2 summarizes the data and statistical analysis of correlation between I-BrQ scores, SRS-22 scores and Cobb degree value at time of questionnaire filling. Global I-BrQ correlates strongly with SRS-22 (r = 0.826; p < 0.001). Also sub scores from each I-BrQ domain strongly correlated with the single domain scores of SRS-22, except for the BrQ Physical Functioning and BrQ School Activity that had an extremely weak and non significative correlation with Management Satisfaction/Dissatisfaction. Only 2 I-BrQ sub scores weakly inversely correlated with Cobb degree value.

**Table 2:** Correlations between the I-BrQ scores and the SRS-22 scores and Cobb degree values

	**Total Score SRS-22**	**SRS-22 Function**	**SRS-22 Pain**	**SRS-22 Mental Health**	**SRS-22 Self image**	**SRS-22 Management Satisfaction/ dissatisfaction**	**Cobb (at quest time)**
**Total Score BrQ**	R = .826	R = .596	R = .702	R = .741	R = .611	R = .432	R = −.189
	P = .000*	P = .000 *	P = .000*	P = .000*	P = .000*	P = .000*	P = .050
**BrQ General Health perception**	R = .639	R = .421	R = .576	R = .582	R = .374	R = .269	R = −.136
	P = .000*	P = .000*	P = .000*	P = .000*	P = .000*	P = .005*	P = .160
**BrQ Physical Functioning**	R = .432	R = .375	R = .483	R = .372	R = .294	R = .106	R = −.037
	P = .000*	P = .000*	P = .000*	P = .000*	P = .002*	P = .275	P = .707
**BrQ Emotional Functioning**	R = .666	R = .419	R = .495	R = .561	R = .618	R = .444	R = −.139
	P = .000*	P = .000*	P = .000*	P = .000*	P = .000*	P = .000*	P = .150
**BrQ Self-esteem and aestetics**	R = .663	R = .291	R = .413	R = .665	R = .596	R = .488	R = −.084
	P = .000*	P = .002^*^	P = .000*	P = .000*	P = .000*	P = .000*	P = .386
**BrQ Vitality**	R = .666	R = .483	R = .508	R = .637	R = .459	R =. 402	R = −.130
	P = .000*	P = .000*	P = .000*	P = .000*	P = .000*	P = .000*	P = .180
**BrQ School Activity**	R = .384	R = .379	R = .317	R = .310	R = .268	R = .141	R = −.014
	P = .000*	P = .000*	P = .001*	P = .001*	P = .005*	P = .144	P = .888
**BrQ Bodily Pain**	R = .569	R = .401	R = .697	R = .506	R = .293	R = .199	R = −.212
	P = .000*	P = .000*	P = .000*	P = .000*	P = .002*	P = .039*	P = .027*
**BrQ Social Functioning**	R = .622	R = .540	R = .399	R = .531	R = .516	R = .352	R = −.192
	P = .000*	P = .000*	P = .000*	P = .000*	P = .000*	P = .000*	P = .046*

* Singnificative value: p < 0.05.

Reproducibility was very good. Measured with Spearman-Brown coefficient (test-retest analysis) the coefficient value was 0.943 (p < 0.05).

## Discussion

During the recent years, as considerable attention was focused on patients’ HRQoL, patient oriented questionnaire have consequently gained great importance being the means by which it is measured and quantified. Disease specific questionnaire have even greater importance, as outcome measures, when referring to a specific pathology. AIS, is a frequent pathology affecting adolescents between 10 and 18 years old. It may seem obvious that treating the disease, with the following improvement of the pathological condition, should immediately translate in a better HRQoL for the patient. However clinical experience, afterwards confirmed by several studies [15-18] showed that brace therapy does have an impact on QoL that must be considered, as it might affect compliance to therapy and healing course. AIS treated with conservative bracing treatment has an important impact on self-image and QoL in patients that are already in emotionally fragile years of their lives regarding self-esteem and social relationships.

A disease-specific questionnaire for measuring QoL in adolescents with idiopathic scoliosis, SRS-22, was already available but it does not include any specific questions on bracing therapy and how it affects QoL. Brace Questionnaire was firstly developed in order to have a doubly specific tool to assess QoL in AIS children treated with a brace.

In order to be used in countries different from the one where it was developed, it is important to adapt it to the culture and language in which it is intended to be used through a validation process [27].

At the end of our study we can asses that:

1. Validation process and particularly construct validity showed a strong positive correlation between I-BrQ total score and SRS-22 total score.

2. A similar strong correlation was found also between most of the single domain sub scores of I-BrQ and SRS-22.

3. Curve magnitude at time of questionnaire filling (measured with Cobb degree scale) correlates with only 2 I-BrQ sub scores. This was an awaited result, as it has been described by other authors [8]: this result suggests that it is not the entity of the curve (being this a sample affected by mild scoliosis) to deteriorate QoL but wearing the brace itself.

In fact, a research of Kotzicki et al., using BSSQ, showed that conservatively treated patients experienced greater stress associated with brace wear (BSSQ-B = 9) than deformity (BSSQ-D = 18) [28].

## Conclusions

Trans-cultural validation in Italian language showed the validity and reliability of the I-BrQ. We hope that trans-cultural validation will be performed in other languages because the availability of the tool as valid outcome measure in different countries may be useful to compare results from larger sample and stimulate secondary publications. It is crucial to acquire more evidence in this field. Moreover I-BrQ should be routinely implemented during brace treatment to monitoring QoL and to provide psychological support if needed. This approach may increase the compliance to treatment, which is instrumental for a successful outcome.

## Competing interests

The authors declare that they have no competing interests.

## Authors’ contributions

All authors read and approved the final manuscript.

## Supplementary Material

Additional file 1 Italian version of Brace Questionnaire (I-BrQ). Click here for file
