# Supplementary material for: Validation of Italian version of Brace Questionnaire (BrQ)
Source: Scoliosis. 2013 Aug 20;8:13. doi: 10.1186/1748-7161-8-13 (PMC3765932; doi:10.1186/1748-7161-8-13)
Supplement: Additional file 1 — Italian version of Brace Questionnaire (I-BrQ). [file 1748-7161-8-13-S1.doc]

***Additional file 1 – Italian version of Brace Questionnaire (I-BrQ)***

***BRACE QUESTIONNAIRE (I-BrQ)***

Nome…………………………. Cognome………………………….

Queste domande valutano come ti senti quando indossi il corsetto. Per favore leggi attentamente questo questionario e rispondi in maniera veritiera. Dopo la valutazione di questo questionario per noi sarà più facile darti consigli per migliorare il trattamento.

- **percezione generale della propria salute**

**Durante gli ultimi 3 mesi**

- - 1. *il corsetto ti ha fatto stare male*
- sempre
- la maggior parte del tempo
- qualche volta
- quasi mai
- mai
  1. *hai avuto paura che la tua schiena potesse peggiorare*
- sempre
- la maggior parte del tempo
- qualche volta
- quasi mai
- mai
- **funzione fisica:**

**Durante gli ultimi 3 mesi mentre portavi il corsetto**

1. *ti sei sentito/a stanca mentre camminavi*

- sempre
- la maggior parte del tempo
- qualche volta
- quasi mai
- mai

1. *non eri in grado di correre*

- sempre
- la maggior parte del tempo
- qualche volta
- quasi mai
- mai

1. *riuscivi ad indossare il corsetto senza l’aiuto di nessuno*

- sempre
- la maggior parte del tempo
- qualche volta
- quasi mai
- mai

1. *riuscivi a togliere il corsetto senza l’aiuto di nessuno*

- sempre
- la maggior parte del tempo
- qualche volta
- quasi mai
- mai

1. *non hai potuto mangiare bene*

- sempre
- la maggior parte del tempo
- qualche volta
- quasi mai
- mai

1. *non hai potuto dormire bene*

- sempre
- la maggior parte del tempo
- qualche volta
- quasi mai
- mai

1. *non hai potuto respirare bene*

- sempre
- la maggior parte del tempo
- qualche volta
- quasi mai
- mai
- **funzione emozionale:**

**Durante gli ultimi 3 mesi**

1. *il corsetto ti ha fatto sentire nervoso/a*

- sempre
- la maggior parte del tempo
- qualche volta
- quasi mai
- mai

1. *ti sei sentito/a preoccupato/a a causa del corsetto*

- sempre
- la maggior parte del tempo
- qualche volta
- quasi mai
- mai

1. *ti sei sentito/a felice*

- sempre
- la maggior parte del tempo
- qualche volta
- quasi mai
- mai

1. *hai creduto che la tua vita sarebbe stata migliore se non avessi portato il corsetto*

- sempre
- la maggior parte del tempo
- qualche volta
- quasi mai
- mai

1. *hai ritenuto che il trattamento con il corsetto sia stato utile*

- sempre
- la maggior parte del tempo
- qualche volta
- quasi mai
- mai
- **percezione estetica del proprio corpo:**

**Durante l’ultimo mese**

1. *ti sei sentito/a fiero/a di te stesso*

- sempre
- la maggior parte del tempo
- qualche volta
- quasi mai
- mai

1. *eri soddisfatto/a del tuo corpo*

- sempre
- la maggior parte del tempo
- qualche volta
- quasi mai
- mai
- **vitalità:**

**Durante l’ultimo mese**

1. *ti sei sentito/a forte e pieno/a di energia*

- sempre
- la maggior parte del tempo
- qualche volta
- quasi mai
- mai

1. *ti sei sentito/a stanco/a ed esausto/a a causa del corsetto*

- sempre
- la maggior parte del tempo
- qualche volta
- quasi mai
- mai
- **attività scolastica:**

**Durante l’ultimo mese a causa del corsetto**

1. *hai avuto difficoltà con le tue lezioni*

- sempre
- la maggior parte del tempo
- qualche volta
- quasi mai
- mai

1. *sei stato/a assente da scuola*

- sempre
- la maggior parte del tempo
- qualche volta
- quasi mai
- mai

1. *hai trovato difficile prestare attenzione in classe*

- sempre
- la maggior parte del tempo
- qualche volta
- quasi mai
- mai
- **dolore:**

**Durante l’ultimo mese mentre portavi il corsetto**

1. *hai preso medicine per il dolore*

- sempre
- la maggior parte del tempo
- qualche volta
- quasi mai
- mai

1. *hai avuto dolore durante la notte*

- sempre
- la maggior parte del tempo
- qualche volta
- quasi mai
- mai

1. *hai avuto dolore mentre camminavi*

- sempre
- la maggior parte del tempo
- qualche volta
- quasi mai
- mai

1. *hai avuto dolore mentre eri seduto/a*

- sempre
- la maggior parte del tempo
- qualche volta
- quasi mai
- mai

1. *hai avuto dolore quando salivi le scale*

- sempre
- la maggior parte del tempo
- qualche volta
- quasi mai
- mai

1. *hai sentito del formicolio alle braccia o alle gambe*

- sempre
- la maggior parte del tempo
- qualche volta
- quasi mai
- mai
- **attività sociale:**

**Durante l’ultimo mese a causa del corsetto**

1. *non sei potuto/a uscire con i tuoi amici*

- sempre
- la maggior parte del tempo
- qualche volta
- quasi mai
- mai

1. *i tuoi amici hanno provato compassione per te*

- sempre
- la maggior parte del tempo
- qualche volta
- quasi mai
- mai

1. *ti sei sentito/a diverso/a dai tuoi compagni*

- sempre
- la maggior parte del tempo
- qualche volta
- quasi mai
- mai

1. *hai avuto problemi con la tua famiglia*

- sempre
- la maggior parte del tempo
- qualche volta
- quasi mai
- mai

1. *hai creduto che la relazione con la tua famiglia o con i tuoi amici sarebbe stata migliore se tu non avessi portato il busto*

- sempre
- la maggior parte del tempo
- qualche volta
- quasi mai
- mai

1. *sei stato/a a casa perché ti vergognavi*

- sempre
- la maggior parte del tempo
- qualche volta
- quasi mai
- mai

1. *hai portato vestiti speciali*

- sempre
- la maggior parte del tempo
- qualche volta
- quasi mai
- mai
